# Skin-Brain Axis: neural pathways in acupuncture treatment

**DOI:** 10.1186/s13020-025-01213-y

**Published:** 2025-10-07

**Authors:** Teng He, Yuanjia Zheng, Jinglan Yan, Yucen Xia, Bokai Wang, Zhen Zhang, Zuoxiang Shang, Kangshuai Li, Bodong Liu, Ning Weng, Yongjun Chen

**Affiliations:** 1 Institute of Acupuncture and Moxibustion, Shandong University of Traditional Chinese Medicine, 4655 University Road, Jinan, 250355 People’s Republic of China; 2 Shandong Key Laboratory of Innovation and Application Research in Basic Theory of Traditional Chinese Medicine, Shandong University of Traditional Chinese Medicine, Jinan, 250355 People’s Republic of China; 3 Key Laboratory of Traditional Chinese Medicine Classical Theory, Ministry of Education, Shandong University of Traditional Chinese Medicine, Jinan, 250355 People’s Republic of China; 4 School of Chinese Medicine, Shandong University of Traditional Chinese Medicine, Jinan, 250355 People’s Republic of China; 5 Department of Traditional Chinese Medicine, Shandong Mental Health Center, Shandong University, Jinan, Shandong People’s Republic of China

**Keywords:** Skin-Brain Axis, Acupuncture, Neural pathways

## Abstract

The “Skin-Brain Axis” hypothesis posits that the skin contains a dense network of nerve endings, neurotransmitters, and neuropeptide receptors capable of detecting tissue damage with high precision and relaying signals to the brain through sensory neurons. Research indicates that therapies involving body surface stimulation, such as acupuncture, modulate brain function. However, there is a paucity of reviews detailing the mechanisms or pathways underlying these therapeutic interventions. This review digs into the neurobiological substrates of acupuncture’s efficacy, focusing on three pivotal components: the activation of skin at acupoints, the conduction of peripheral nerve signals, and the subsequent central nervous system responses.

**Graphical Abstract:**

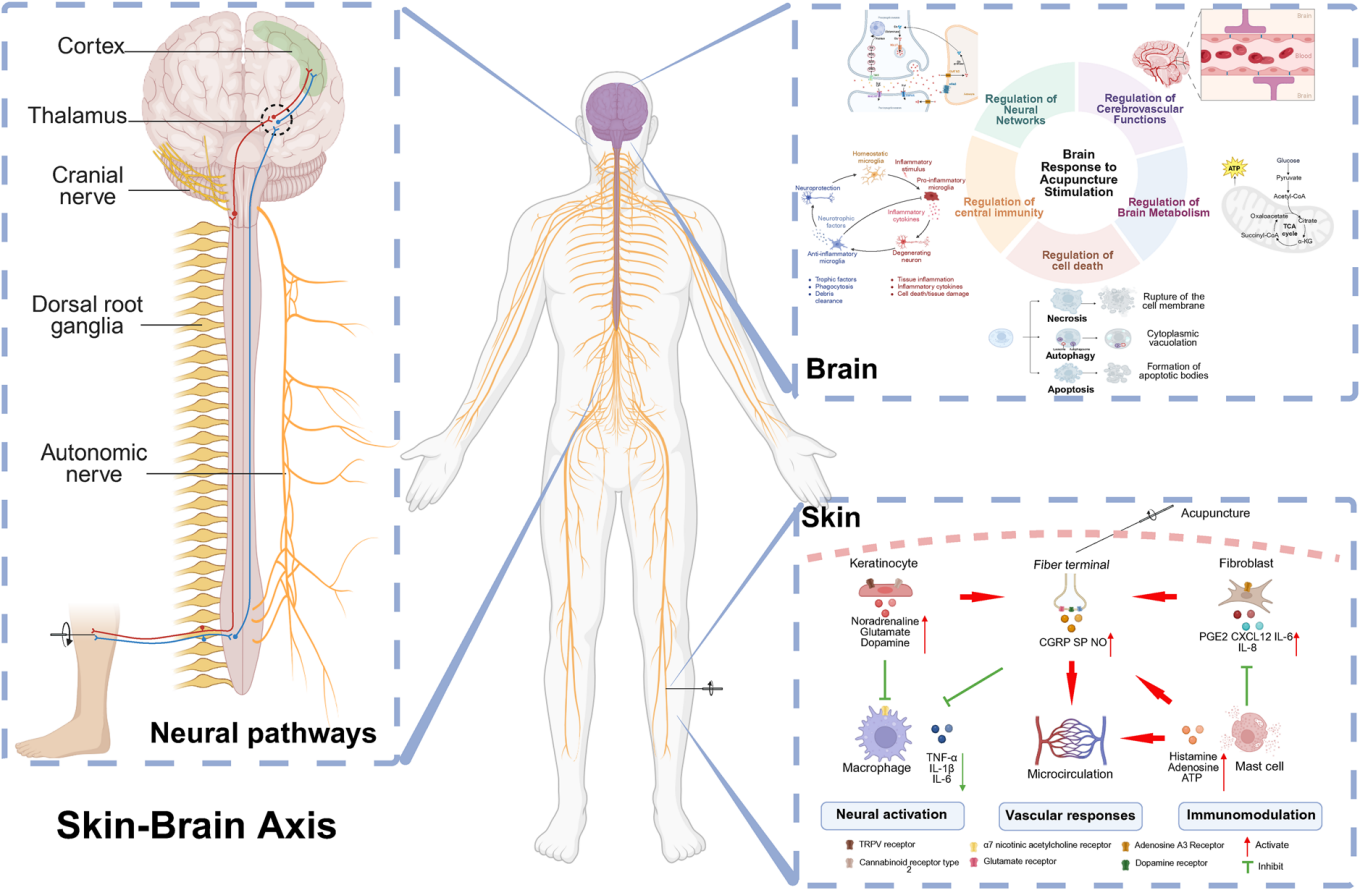

## Introduction

The skin, a dynamic reflector of the brain’s stress responses and a primary gateway for sensory perception of the external environment [1], shares a profound developmental connection with the central nervous system (CNS), both originating from the ectodermal layer during embryonic development [2, 3]. This intimate relationship is encapsulated in the “Skin-Brain Axis,” which portrays the complex interplay between the skin and the brain, facilitated through the nervous, endocrine, and immune systems [4, 5]. The skin’s rich network of neural endings and its capacity to detect and transmit tissue damage signals via sensory nerves, through the presence of neurotransmitter and neuropeptide receptors, underscores its sophisticated sensory and neural capabilities.

Acupuncture harnesses the skin’s sensory and neural attributes by stimulating specific acupoints to regulate physiological functions across various organ systems. Randomized controlled trials have attested to the therapeutic efficacy of acupuncture in addressing conditions such as post-stroke motor aphasia [6], postoperative pain following cesarean delivery [7], and migraine headaches [8]. Nonetheless, the detailed biological underpinnings of these therapeutic outcomes are not yet fully understood. This review assembles evidence that focuses on the “Skin-Brain Axis”, which digs into three interwoven mechanisms: the activation of skin at acupoints, the conduction of peripheral neural signals, and the subsequent central brain regions’ responses to acupuncture stimulation.

## Physiological basis of acupoint microenvironments

### Anatomical architecture of acupoint tissues

Acupoints exhibit multilayered anatomical complexity, comprising the epidermis, dermis, subcutaneous tissue, musculature, and associated neurovascular bundles (nerve fibers, blood vessels, and lymphatic channels). Notably, the acupoint epidermis exhibits reduced thickness, while the dermal layer harbors dense capillary networks (diameter: 5–400 μm) and free nerve endings enriched with mechanosensitive receptors (e.g., Transient receptor potential vanilloid 1/3/4; TRPV1/3/4) [9]. Approximately 80% of acupoints localize to subcutaneous connective tissue, where collagen/elastic fibers and fascial structures form a biomechanical transduction matrix [9]. Acupoints demonstrate elevated vascular density, featuring superficial capillary glomeruli and deep longitudinal vascular bundles, with lymphatic vessels paralleling nerve tracts [10]. Neural fiber density at acupoints exceeds non-acupoint regions by 1.4-fold, encompassing Aβ (large-diameter myelinated), Aδ (thinly myelinated), and C-fibers (unmyelinated nociceptors) [11]. Mechano-/chemo-receptor diversity is pronounced, with free nerve endings, corpuscular receptors (e.g., Ruffini corpuscles), and muscle spindles concentrated at acupoints, many of which colocalize with major nerve trunks or plexuses [12, 13].

### Cellular heterogeneity in acupoint microenvironments

Acupoint regions exhibit high cellular heterogeneity, primarily comprising four functional categories. Classic immune cell populations: mast cells show a 55% higher density in acupoint regions compared to non-acupoint areas, with synaptic-like connections observed between mast cells and nerve terminals [14]. M2-polarized macrophages promote tissue repair through secretion of interleukin-10 (IL-10) and transforming growth factor-beta [15]. Barrier and immune regulatory cells: keratinocytes mediate mechanotransduction via TRPV1/3/4 receptors, secrete neurotransmitters such as glutamate and dopamine to activate C-fiber terminals, and maintain immune homeostasis through cortisol release via the hypothalamic–pituitary–adrenal (HPA) axis [16]. Fibroblasts sense shear stress through the ROCK signaling pathway and recruit immune cells by secreting chemokines like interleukin-6(IL-6) and C-X-C motif chemokine ligand 12(CXCL12) [17]. Neural-related cell populations: peptidergic and non-peptidergic subtypes of C-fiber nociceptors synergistically transmit nociceptive signals [18]. Silent nociceptors acquire mechanical sensitivity under inflammatory stimulation [19]. Satellite glial cells mediate neuronal synchronization through gap junctions [20]. Vascular-immune interface cells: endothelial cells regulate microcirculation by releasing nitric oxide (NO) and endothelin, and guide immune cell migration via secretion of CXCL8 and monocyte chemoattractant protein-1(MCP-1) [21].

The collective presence and multidirectional interactions of these previously mentioned cellular elements constitute the unique microenvironment of acupoints. As the primary targets for acupuncture stimulation, the functional cells and their associated biochemical responses within this microenvironment represent the essential material basis underpinning the therapeutic effects of acupuncture.

## Multisystem interaction mechanisms underlying acupuncture activation

The acupoint microenvironment exhibits structural specialization for mechanotransduction, featuring high-density mechanoreceptors, amplified vascular networks, and immune cell clusters. This unique architecture confers specific responsiveness to acupuncture’s mechanical stimuli, initiating integrated neuro-immune-vascular cascades that underpin therapeutic efficacy. Specifically, these structural specializations (coupled neural-stromal interfaces and neuro-immune-vascular triads) enable needle manipulation (lift/thrust/rotation) to generate spatially directional biomechanical stress fields. These fields are transduced into biochemical signals via strain-activated cellular responses, a process essential for activating the coordinated neuro-immune-vascular networks mediating acupuncture efficacy.

### Neural activation pathways

The transduction of acupuncture-induced mechanical stimuli into complex biological signals is mediated by an integrated multicellular mechanosensory complex at acupoints, comprising peripheral neurons, keratinocytes, and fibroblasts. Mechanistic investigations reveal that collagen fiber deformation caused by needle lifting/thrusting and rotation directly stimulates Aδ and C fiber terminals [22]. Clinical studies further demonstrate that low-frequency (1–10 Hz) and high-frequency (50–100 Hz) stimulation selectively activate large-diameter Aβ fibers and small-diameter Aδ/C fibers, respectively, eliciting characteristic numbness/swelling or soreness/pain sensations. These responses correlate significantly with the clinical “deqi” effect [23–25]. Notably, the analgesic effects of Zusanli (ST36) and cortical blood flow restoration induced by Shenting (GV24) are completely abolished by local lidocaine pretreatment, confirming the central role of neural transmission [26–28]. This definitive blockade confirms that peripheral sensory nerve fibers play a critical role in detecting acupuncture stimulation signals. Beyond neuronal pathways, emerging evidence highlights the collaborative roles of non-neuronal cells (keratinocytes and fibroblasts) in signal amplification. Keratinocytes at acupoints specifically express mechanosensitive receptors such as TRPV1/3/4, which respond to acupuncture-induced stress, triggering glutamate and dopamine release to directly activate adjacent nerve terminals [29–32]. What’s more, keratinocytes participate in neural regulation via multimodal mechanisms: beyond basic neurotransmitter secretion, acupuncture stimulation upregulates their surface cannabinoid type 2 receptor expression, promoting endogenous opioid peptide release to directly activate primary sensory neurons [33]. Fibroblasts serve as key mechanotransducers in neural activation. Acupuncture rotation manipulation induces co-linear alignment between fibroblasts and collagen fibers in subcutaneous connective tissue, resulting in doubled cellular cross-sectional area and shortened protrusions. This mechanical deformation activates the Rho-associated protein kinase (ROCK) pathway, promoting the release of pro-inflammatory factors including IL-6 and CXCL12, as well as prostaglandin E2 (PGE2) [34, 35]. In vitro experiments confirm that mechanical stress stimulation elevates adenosine levels in fibroblasts, facilitating cell proliferation through A3 receptor-mediated mitogen-activated protein kinase signaling cascades [17]. Together, these non-neuronal cells and nerve fibers collectively constitute the “neural acupuncture unit”, transforming mechanical stimuli into cross-system biological signals through cascading releases of neurotransmitters, inflammatory factors, and immune regulatory factors [36].

### Immune regulatory network

Acupuncture triggers an innate immune response within the acupoint microenvironment by activating resident immune cells and modulating their secretory profiles. Mast cells, acting as core sensors and effectors for acupuncture stimulation at acupoints, exhibit significantly higher density within acupoint regions compared to non-acupoint regions. They form a tight spatial structure with collagen fibers, microvessels, and nerve endings—termed the “collagen fiber-mast cell-nerve” complex—constituting the biological foundation for acupuncture stimulation [37]. Under pathological conditions (e.g., arthritis), the expression of local chemokines (such as MCP-1) at acupoints is upregulated, promoting mast cell migration towards the acupoints and further increasing their density and activity [38]. The mast cell membrane expresses various mechanosensitive channels (e.g., TRPV2, TRPV4, chloride channels). The lifting-thrusting and twisting manipulations of acupuncture needles apply mechanical stress to mast cells indirectly by stretching collagen fibers, thereby activating these channels [37, 39]. Gene knockout experiments demonstrate that TRPV2 deficiency significantly suppresses acupuncture-induced mast cell degranulation and its analgesic effect [39]. Activated mast cells release multiple mediators. Released adenosine triphosphate (ATP) and histamine both activate local nerve endings, inhibiting pain signal transmission [39, 40]. Mast cell-derived 5-Hydroxytryptamine (5-HT) promotes ATP release via the 5-HT1A receptor, amplifying the acupuncture signal [41]. Thus, mast cells play an indispensable, pivotal role in sensing acupuncture stimulation, triggering local neuro-immune responses, and ultimately mediating acupuncture effects such as analgesia. Moreover, cellular components such as keratinocytes and fibroblasts constitutively maintain immune homeostasis within the distinct acupoint microenvironment through coordinated cytokine release. Needle stimulation induces keratinocytes to secrete HPA axis-related hormones (corticotropin-releasing hormone, adrenocorticotropic hormone, cortisol), which suppress the activity of pro-inflammatory transcription factors such as nuclear factor kappa B (NF-κB) while upregulating the expression of anti-inflammatory factors including IL-4 and IL-10, thereby achieving localized immune homeostasis regulation [42, 43]. Fibroblast-derived IL-6 and PGE2 not only contribute to the construction of a local inflammatory microenvironment but also regulate distal immune cell phenotype transformation via the circulatory system, such as promoting the polarization of pro-inflammatory macrophages toward anti-inflammatory subtypes [15, 17]. Experimental data demonstrate that electroacupuncture significantly inhibits local PGE2 release, thereby alleviating neuropathic pain [44]. These findings collectively suggest that mediators released by mast cell, keratinocytes, and fibroblasts degranulation constitute the molecular foundation of immune regulation.

### Vascular dynamic responses

The core mechanism underlying acupuncture’s dynamic regulation and homeostatic maintenance of local and systemic blood circulation resides in the coordinated interaction between neural signaling triggered by mechanical stimulation and diverse bioactive mediators. Although needle diameters (0.25–0.9 mm) typically exceed cutaneous microvascular dimensions (70–120 μm), biochemical signals (e.g., CGRP, NO) induced by mechanical stimulation remain the core mechanism mediating vasodilation [45, 46]. Needle manipulation (e.g., dry needling) induces the release of vasoactive substances (CGRP, substance P), triggering micro arteriolar dilation via axon reflexes, which significantly enhances tissue blood flow and oxygen saturation [47, 48]. Advanced studies reveal that acupuncture stimulation increases local NO production, optimizing microcirculation through vasodilation and enhancing endothelial function [49, 50]. This process is closely associated with sympathetic reflexes: the “deep sympathetic reflex theory in vascular walls” proposed by Xu et al. demonstrates that mechanical stimulation from acupuncture triggers neural reflexes to dynamically regulate collateral blood flow distribution [51]. Beyond neural mechanisms, fibroblasts and keratinocytes also play significant roles in acupuncture’s dynamic vascular regulation. Under mechanical stimulation from needling, fibroblasts contribute indirectly to vascular dynamics: released IL-6 and PGE2 modulate vascular permeability, while extracellular matrix remodeling following mechanical deformation may optimize local hemodynamics [17]. Concurrently, keratinocytes maintain endothelial functional stability by secreting HPA axis hormones (e.g., cortisol) to suppress vascular inflammatory responses [43]. In summary, acupuncture achieves dynamic optimization and homeostasis maintenance of the microcirculatory system through a multitiered synergistic mechanism involving neural reflexes (axon/sympathetic reflexes), biochemical signal mediation (NO/ATP), cytokine regulation (IL-6/PGE2), and endocrine modulation (cortisol).

**Fig. 1:**
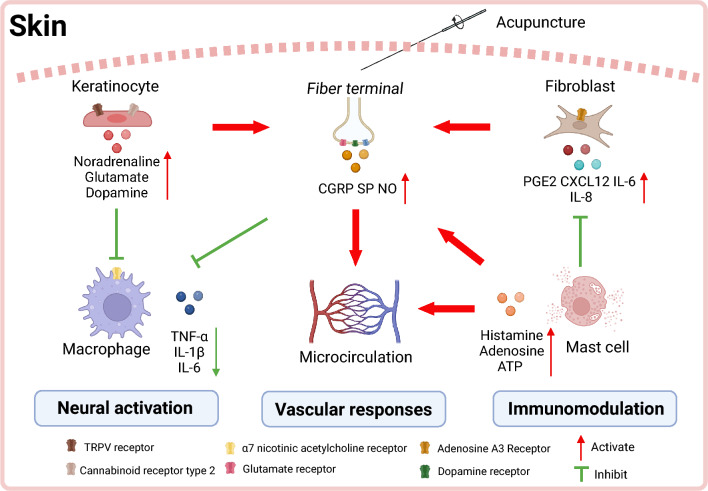
The diagram elucidates activation of acupoint skin by acupuncture. (Created with BioRender.com)

Collectively, acupoint activation involves responses across three subsystems: neural transduction via multicellular mechanosensory complexes, immune reconfiguration through mast cell-keratinocyte-fibroblast interactions, and hemodynamic optimization mediated by neurovascular coupling (Fig. 1). This integrated peripheral response initiates peripheral nervous system (PNS) sensitization through neural pathways. Mechano-sensitive C/Aδ-fiber activation triggers action potentials propagating to dorsal root ganglia (DRG). These processes establish the foundation for CNS modulation discussed in Chapter 3.

## Activation of the PNS: a necessary pathway for acupuncture efficacy

Neural signals generated within the acupoint microenvironment (detailed in Sects. “Neural activation pathways”–“Immune regulatory network”) are transmitted to the spinal cord via peripheral sensory afferent pathways, where they undergo primary integration and modulation. This conduction process relies on the topographically specific connection patterns established between the DRG and spinal cord segments corresponding to distinct somatic regions [52, 53]. Modern neural tracing techniques have precisely verified this principle of somatotopic organization, revealing that stimulation of different acupoints specifically activates corresponding spinal cord segments: acupoints in the head and face correspond to trigeminal ganglion innervation [54]; upper limb acupoints correspond to DRG within segments C3-T2 [55–58]; lower limb acupoints correspond to DRG within segments T8-L6 [56, 59–65]; and trunk acupoints correspond to DRG within segments T8-L8 [58, 66, 67] (See Table 1 for details). The necessity of these pathways has been confirmed by intervention experiments: for instance, surgical transection of the common peroneal nerve or subdiaphragmatic vagus nerve completely abolishes the analgesic and anti-inflammatory effects of acupuncture at Zusanli (ST36) [68, 69], confirming that peripheral nerves are the essential conduits for signal transmission to the spinal cord.

**Table 1:** Presents a summary of the tracing results for body surface acupuncture points and their corresponding upstream neural ganglia

Acupoint	Localization	Tracer	upstream neural ganglia	Animal
Yangbai (GB14)	On the head, 1 B-cun is superior to the eyebrow, directly superior to the center of the pupil	CTB	TG, DRG C2-C4	Rat [54]
Sibai (ST2)	On the face, in the infraorbital foramen	CTB	TG, DRG C2-C4	Rat [54]
Jiache (ST6)	On the face, one fingerbreadth (middle finger) is anterosuperior to the angle of the mandible	CTB	TG, DRG C2-C4	Rat [54]
Hegu (LI4)	On the dorsum of the hand, radial to the midpoint of the second metacarpal bone	CTB	DRG C5-T1	Rat [72]
Quchi (LI11)	On the lateral aspect of the elbow, at the midpoint of the line connecting LU5 with the lateral epicondyle of the humerus	CTB	DRG C5-T1	Rat [55]
Neiguan (PC6)	On the anterior aspect of the forearm, between the tendons of the palmaris longus and the flexor carpi radialis, 2 B-cun proximal to the palmar wrist crease	HRP	DRG C5-T1	Rat [56, 57]
Shenmen (HT7)	On the anteromedial aspect of the wrist, radial to the flexor carpi ulnaris tendon, on the palmar wrist crease	HRP	DRG T2-T6	Rat [57]
Tianshu (ST25)	On the upper abdomen, 2 B-cun lateral to the center of the umbilicus	CTB	DRG T8-L1	Rat [58]
Futu (ST32)	On the anterolateral aspect of the thigh, on the line connecting the lateral end of the base of the patella with the anterior superior iliac spine, 6 B-cun superior to the base of the patella	HRP	DRG T8-L5	Rat [56]
Weishu (BL21)	In the upper back region, at the same level as the inferior border of the spinous process of the 12th thoracic vertebra (T12), 1.5 Bcun lateral to the posterior median line	HRP	DRG T9-T12	Rat [66]
Zusanli (ST36)	On the anterior aspect of the leg, on the line connecting ST35 with ST41, 3 B-cun inferior to ST35	CTB	DRG L3-L6	Rat [59]

Ququan (LR8)	On the medial aspect of the knee, in the depression medial to the tendons of the semitendinosus and the semimembranosus muscles, at the medial end of the popliteal crease	CTB	DRG L1-L6	Rat [60]
Weizhong (BL40)	On the posterior aspect of the knee, at the midpoint of the popliteal crease	CTB	DRG L2-L6	Rat [61]
Jinggu (BL64)	On the lateral aspect of the foot, distal to the tuberosity of the fifth metatarsal bone, at the border between the red and white flesh	CTB	DRG L3-L6	Rat [62]
Dazhong (KI4)	On the medial aspect of the foot, posteroinferior to the medial malleolus, superior to the calcaneus, in the depression anterior to the medial attachment of the calcaneal tendon	CTB	DRG L3-L6	Rat [62]
Taichong (LR3)	On the dorsum of the foot, between the first and second metatarsal bones, in the depression distal to the junction of the bases of the two bones, over the dorsal pedal artery	CTB	L3-L5	Rat [60]
Yongquan (KI1)	On the sole, in the deepest depression of the sole when the toes are flexed	CTB	DRG L3-L6	Rat [64]
Taixi (KI3)	On the posteromedial aspect of the ankle, in the depression between the prominence of the medial malleolus and the calcaneal tendon	HRP	DRG L5 > L4 > L6	Rat [63]
Chengshan (BL57)	On the posterior aspect of the leg, at the connecting point of the calcaneal tendon with the two muscle bellies of the gastrocnemius muscle	CTB	DRG L4-L5	Rat [65]
Qimen (LR14)	In the anterior thoracic region, in the sixth intercostal space, 4 B-cun lateral to the anterior median line	HRP	DRG L5-L8	Cat [67]

*CTB* cholera toxin subunit B, *HRP* horse radish peroxidase, *TG* trigeminal ganglion, *DRG* Dorsal root ganglion

Within the spinal cord, acupuncture signals are transmitted via the PNS—encompassing spinal nerves (for somatosensory and motor conduction in the limbs/trunk), cranial nerves (for functional regulation of the head and face), and autonomic pathways (for visceral homeostasis regulation) [70]. These input signals are subsequently projected to higher centers via ascending tracts, initiating further processing for nociception, immune responses, and autonomic regulation.

### Activation of spinal nerves

Extensive research demonstrates that acupuncture techniques, including manual acupuncture and transcutaneous electrical nerve stimulation (TENS), exert significant analgesic and neuromodulatory effects by specifically activating distinct classes of afferent nerve fibers (e.g., A-fibers) and regulating the activity, receptor expression, and key molecular pathways in DRG sensory neurons [71]. The following studies elucidate these underlying mechanisms. Under normal physiological conditions, stimulation of Zusanli (ST36) at 1 Hz, accompanied by manipulation techniques like twisting, lifting, and thrusting, maximally induces discharge in spinal dorsal horn neurons, demonstrating effective nerve activation [73]. Low-frequency percutaneous electrical stimulation (2 Hz) applied to Shuigou (GV26), Yanglingquan (GB34), and Zusanli (ST36) enhances μ-opioid receptor expression in the L3–L5 DRG, effectively reducing hyperalgesia in neuropathic pain rat models, whereas high-frequency stimulation (100 Hz) does not yield similar results [74]. Acupuncture at Zusanli (ST36) also decreases the action potential threshold of L4–L6 DRG neurons via A-type nerve fibers, alleviating oxaliplatin-induced neuropathic pain [75]. Electroacupuncture (EA) at Liangqiu (ST34) specifically activates A-type nerve fibers, inhibiting abnormal electromyographic activity due to muscle inflammation and mitigating inflammatory muscle pain by stimulating low-threshold mechanoreceptor neurons in the L4–L6 DRG [76]. Additionally, EA at Zusanli (ST36) and Kunlun (BL60) suppresses TRPV1 receptor activity in sensory neurons of the L4–L6 DRG, offering relief from paclitaxel-induced peripheral neuropathy [77]. Using transgenic Rosa26-GCaMP mice, studies have shown increased neuronal activity in the L6 DRG following colon distension or EA at Dachangshu (BL25), emphasizing its direct effect on nerve function [78]. In collagen-induced arthritis pain models, acupuncture at Zusanli reduces mechanical hypersensitivity by inhibiting sensory neuron activation in the L4–L5 DRG [79]. Similarly, EA at Huantiao (GB30) increases prostatic acid phosphatase (PAP) expression in the L4–L5 DRG, significantly alleviating pain in chronic stretch injury models, and inhibiting PAP expression reverses this analgesic effect, highlighting its importance [80]. Furthermore, EA stimulation at Zusanli (ST36) induces the release of adenosine, modulating nociceptive responses and potentiating neuronal activation within the L4-L6 DRG [81].

In summary, acupuncture, particularly stimulation at specific acupoints with defined parameters, precisely modulates DRG sensory neurons. The mechanisms involve activating specific afferent nerve fibers, altering neuronal excitability, regulating the expression and function of key receptors (e.g., μ-opioid, TRPV1), and modulating the expression of critical molecules (e.g., PAP). This effectively suppresses the generation or transmission of nociceptive signals, ultimately achieving analgesia and neural functional regulation.

### Activation of autonomic nerves

Acupuncture exerts precise regulatory effects on distinct branches of the autonomic nervous system through specific stimulation parameters, playing a pivotal role in neuro-immune modulation. EA at Zusanli (ST36) with a low intensity of 0.5 mA activates the “vagus nerve–adrenal neuropeptide Y-positive medullary cell” pathway, which effectively suppresses systemic inflammation. In contrast, high-intensity EA at Tianshu (ST25) with 5 mA stimulates NPY-positive sympathetic neurons that project to immune organs, such as the spleen, thereby inhibiting inflammation [82, 83]. EA at Shangjuxu (ST37) increases vagal nerve electrophysiological activity within the solitary nucleus, reinforcing its role in autonomic modulation [84]. Auricular acupuncture has demonstrated the ability to restore autonomic balance in rat models with autonomic dysfunction, further illustrating its therapeutic potential [85]. Additionally, EA at Jianshi (PC5) and Neiguan (PC6) reduces visceral sympathetic excitatory reflexes induced by bradykinin in the gallbladder, underlining its capacity to modulate autonomic nervous system responses [86]. Thus, by dynamically balancing sympathetic activity and parasympathetic (vagal) tone, acupuncture achieves targeted suppression of systemic inflammatory responses and homeostatic maintenance of visceral function.

### Activation of cranial nerves

Acupuncture effectively mitigates craniofacial hyperalgesia and central nervous system dysfunction through its targeted modulation of key nuclei and molecular pathways within the trigeminal neural circuitry. Stimulation at Neiting (ST44) has been shown to suppress abnormal neuronal activity in the caudal nucleus of the trigeminal nerve, providing relief from acute dental pulp pain [87]. The application of EA using the olfactory three-needle technique has improved cognitive impairments and enhanced hippocampal synaptic plasticity by stimulating the olfactory nerve, which in turn activated the brain-derived neurotrophic factor (BDNF)/CAMP response element-binding (CREB) pathway and phosphoinositide 3-kinase (PI3K)/protein kinase b (AKT)/mammalian target of rapamycin (mTOR) signaling pathways [88]. Facial acupuncture point stimulation has alleviated ocular surface neuralgia in guinea pigs with dry eye by inhibiting the purinergic receptor P2X3-protein kinase C (PKC) in the trigeminal ganglion and spinal trigeminal nucleus caudalis [89]. Moreover, stimulation at Shuaigu (GB8) has suppressed astrocyte activation and the expression of the pro-inflammatory cytokine interferon-gamma in the spinal trigeminal nucleus, yielding anti-inflammatory and analgesic effects in migraine model rats [90]. Collectively, acupuncture employs a multi-tiered regulatory strategy for trigeminal system modulation.

## Brain responses to acupuncture stimulation

The brain serves as the central command center for controlling and coordinating bodily functions, processing information, and regulating behavior through intricate neuronal networks. Advances in neural circuit research have significantly enhanced our comprehension of the anatomical and functional correlations between body surface acupoints and central brain regions, providing novel insights into the mechanisms by which acupuncture exerts its therapeutic effects.

### CNS as the anatomical basis of acupuncture efficacy

**Fig. 2:**
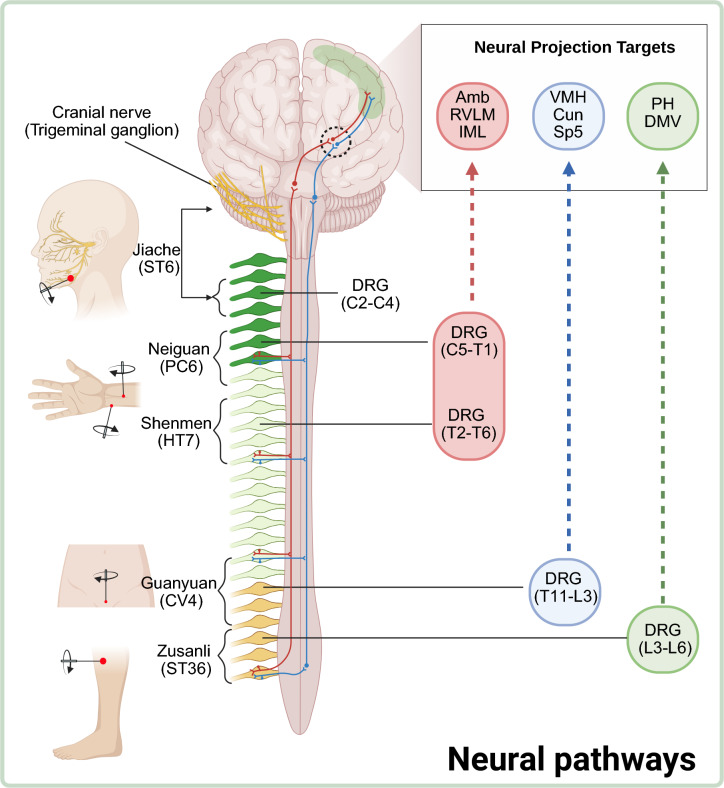
Illustrative diagram showing segmentally organized neural pathways from representative acupoints to central nuclei (Created with BioRender.com). *Amb* nucleus ambiguus, *Cun* cuneate nucleus, *DMV* dorsal motor nucleus of vagus, *DRG* dorsal root ganglion, *IML* intermedilateral nucleus, *PH* paraventricular hypothalamic nucleus, *RVLM* rostral ventrolateral medulla, *Sp5* spinal trigeminal nucleus, *VMH* ventromedial hypothalamic nucleus

State-of-the-art neurobiological methods, such as cross-multilevel synaptic virus tracing, have facilitated direct exploration of the anatomical connections between acupoints and central brain areas. Utilizing pseudorabies virus (PRV) as a neural tracer, injections at specific acupoints like Zusanli (ST36), Guanyuan (CV4), Neiguan (PC6), and Shenmen (HT7) have uncovered unique patterns of viral transfection across various brain regions. For instance, PRV injection at Zusanli (ST36) revealed transfection in areas implicated in autonomic regulation and emotional processing, including the dorsal motor nucleus of the vagus nerve, nucleus of the solitary tract, area postrema, A1 and C1 adrenergic clusters, caudal ventrolateral reticular nucleus, locus coeruleus, multiple hypothalamic nuclei, and the amygdala [91]. In contrast, PRV injections at Neiguan (PC6) and Shenmen (HT7) predominantly targeted regions involved in autonomic regulation, such as the solitary tract nucleus, paraventricular hypothalamic nucleus, A1 and C1 cell clusters, and locus coeruleus. Similarly, PRV injection at Guanyuan (CV4) led to viral transfection in the solitary tract nucleus, cuneate fascicular nucleus, the dorsal motor nucleus of the vagus nerve, and various thalamic and hypothalamic nuclei [92]. These findings underscore the specificity of acupoint-brain interactions and their physiological significance, revealing distinct transmission pathways and transfection patterns that vary by acupoint (See Table 2 for details and Fig. 2). This neuroanatomical evidence lays the groundwork for understanding how acupuncture modulates brain function.

**Table 2:** Anatomical connectivity between body surface acupuncture points and central brain regions

Acupoint	Localization	Tracer	Peripheral neural ganglia	Spinal cord	Central brain regions	Animal
Zusanli (ST36)	On the anterior aspect of the leg, on the line connecting ST35 with ST41, 3 B-cun inferior to ST35	PRV	DRG L3-L6	Lam (IV, V, VII, X), IML	A1; A5; AP; Arc; Amyg; BNST; CGM; CVLRN; DCN; DMV; Gi; LH; LC; PN; PH; PVN; RM; ROb; SN; SubC;	Rat [91]
Guanyuan (CV4)	On the lower abdomen, 3 B-cun inferior to the center of the umbilicus, on the anterior median line	PRV	DRG T11-L3	Thoracic, Lumbar and Sacral spinal segments	Arc; Ctx; Cun; DMV; DPT; Gi; IO; IST; LC; MTrN; Me5; PM; PnC; PCRt; PVH; RM; SN; Sp5; VT; VMH	Rat [92]
Neiguan (PC6) Shenmen (HT7)	Neiguan (PC6): On the anterior aspect of the forearm, between the tendons of the palmaris longus and the flexor carpi radialis, 2 B-cun proximal to the palmar wrist crease Shenmen (HT7): On the anteromedial aspect of the wrist, radial to the flexor carpi ulnaris tendon, on the palmar wrist crease	PRV	Neiguan (PC6): DRG C5-T1 Shenmen (HT7): DRG T2-T6	Neiguan (PC6): LCN, Lam (I–V, VII, X), IML, LSN, Lam (I, II, IV, V, VII–X), Lam (III, IV), Lam (IV, V, VII) Shenmen (HT7): Lam (I, II, IV–VI, X), IML, LSN, Lam (I, II, IV, V, VII–X), Lam (III, IV), Lam (III, IV, V, X)	Neiguan (PC6): A1; A5; Amb; Arc; C1; C3; CGM; CVLRN; IML; LH; LC; LPGi; PVH; PBN; RM; ROb; RP; RVLM; SN; SubC Shenmen (HT7): A1; A5; Amb; Arc; C1; C3; CGM; CVLRN; IML; LC; LPGi; PVH; RM; ROb; RP; RVLM; SN; SubC	Rat [57]

*A1* A1 noradrenergic cells, *A5* A5 cell group, *AP* Area postrema, *Arc* Arcuate nucleus of the hypothalamus, *Amyg* Amygdala, *BNST* Bed nucleus of the stria terminalis, *CGM* Central gray matter, *C1* C1 adrenergic cell group, *C3* C3 adrenergic cell group*, Cun* Cuneate nucleus, *CVLRN* Caudal ventrolateral reticular nucleus, *DCN* Dorsal column nuclei, *DMV* Dorsal motor nucleus of the vagus, *DPT* Dorsal pontine tegmental nucleus, *Gi* Gigantocellular nucleus, *IML* Intermediolateral cell column, *IO* Inferior olivary nucleus, *IST* Interstitial nucleus of the stria terminalis, *LH* Lateral hypothalamic nucleus, *LC* Locus coeruleus, *LPGi* Lateral paragigantocellular reticular nucleus, *MTrN* Main trigeminal nucleus, *Me5* Mesencephalic trigeminal nucleus, *PM* Preoptic magnocellular nucleus, *PnC* Caudal pontine reticular nucleus, PCRt Parvocellular reticular nucleus, *PH* Posterior hypothalamic nucleus, *PN* Pallid nucleus, PVH Paraventricular hypothalamic nucleus, *PBN* Parabrachial nucleus, *RM* Raphe magnus nucleus, *ROb* Raphe obscurus nucleus, *RP* Raphe pallidus nucleus, *RVLM* Rostral ventrolateral medulla*, SN* Solitary nucleus, *Sp5* Spinal trigeminal nucleus, *SubC* Subcoeruleus nucleus, *VT* Ventral thalamic nucleus, *VMH* Ventromedial nucleus, *Lam* Laminae, *IML* Intermediolateral nucleus, *LCN* Lateral cervical nucleus, *LSN* Lateral spinal nucleus

### Dynamic neuromodulation of acupuncture on central brain regions and functional networks

As a cornerstone intervention in traditional medicine, acupuncture has been extensively validated in clinical practice for its therapeutic efficacy. The advent of functional magnetic resonance imaging (fMRI) has offered empirical evidence to decipher the neurobiological mechanisms through which acupuncture modulates brain function. Emerging research demonstrates that acupoint-specific stimulation elicits precise modulation of neural activity and functional connectivity across distributed brain networks, effectively ameliorating pathological states in hypertension, migraines, and sleep disorders. These neuroimaging-driven findings not only delineate the multi-target modulatory effects of acupuncture at the circuit level but also establish a translational bridge for modernizing traditional therapeutic approaches within evidence-based medicine (See Table 3 for details).

1. Hypertension: Unilateral acupuncture at Taichong (LR3) increased the amplitude of low-frequency fluctuation (ALFF) and regional homogeneity (ReHo) in the hypothalamus while enhancing its functional connectivity with the brainstem and cerebellum [93]. Combinatorial stimulation of Taichong (LR3) and Yongquan (KI3) significantly reduced systolic blood pressure (SBP) and diastolic blood pressure (DBP), concurrently activating the prefrontal cortex (Brodmann areas 9, 19, 46), cingulate cortex (Brodmann areas 6, 23, 24, 31), hypothalamic nuclei, and supplementary motor area (Brodmann areas 6) [94].
2. Migraine: Acupuncture at Fengchi (GB20) and Hegu (LI4) modulated the amygdala (AMYG), insula (INS), and mid-cingulate cortex (MCC), strengthening connectivity within multisensory processing regions including the middle temporal gyrus (MTG) and superior temporal gyrus (STG) [95]. Acupuncture robustly activated the right amygdala, left insula, and medial orbitofrontal cortex (ventromedial prefrontal cortex, vmPFC), enhancing pain modulatory network functionality [96]. Combined Taichong (LR3) and Hegu (LI4) stimulation augmented functional coupling between hypothalamic gray matter (ventrolateral periaqueductal gray, vlPAG) and dorsolateral prefrontal cortex (DLPFC), correlating with pain alleviation [97]. Acupuncture reversed functional aberrations in headache-related regions (amygdala, thalamus, anterior cingulate cortex), reducing attack frequency [98].
3. Insomnia: Baihui (GV20) combined with Shenmen (HT7) strengthened functional connectivity among the anterior cingulate cortex (ACC), hippocampus, and amygdala, ameliorating anxiety-related insomnia [99]. Acupuncture at Shenmen (HT7), Sanyinjiao (SP6), and Baihui (GV20) elevated ALFF values in the right cerebellar posterior lobe, bilateral brainstem, and frontoparietal regions, modulating autonomic nervous activity [100]. Treatment normalized aberrant connectivity between the ventromedial hypothalamic nucleus (VMH) and lateral orbitofrontal cortex (LOFC), improving sleep efficiency metrics [101].
4. Post-Stroke Functional Impairment: Jin’s three-needle technique (JTN) activated the cerebellar posterior lobe, inferior parietal lobule, and middle temporal gyrus, facilitating motor control and sensory integration [102]. Acupuncture at Hegu (LI4) and Zusanli (ST36) modulated the sensorimotor network (SMN) and default mode network (DMN), improving limb functional recovery [103]. Stimulation of Neiguan (PC6) and Shuigou (DU26) enhanced connectivity between the motor cortex and SMN, promoting neuroplastic reorganization [104].
5. Cognitive Impairment: Electroacupuncture at Baihui (GV20) and Shenmen (HT7) strengthened hippocampal-DMN connectivity, ameliorating diabetes-associated cognitive impairment [105]. Acupuncture at Taichong (LR3) and Yongquan (KI3) augmented functional coupling between the hippocampus and temporoparietal regions in Alzheimer’s disease (AD) patients [106]. Combinatorial stimulation of Baihui (GV20) and Zusanli (ST36) increased hippocampal volume and DMN synchronization, alleviating subjective cognitive decline (SCD) [107].
6. Metabolic and Pain-Related Disorders: Acupuncture at Tianshu (ST25) and Zusanli (ST36) modulated hypothalamic NPY/AgRP and POMC/CART neuronal populations, inducing appetite suppression [108]. Yanglingquan (GB34) stimulation rebalanced functional connectivity between the ventrolateral periaqueductal gray (vlPAG) and dorsolateral prefrontal cortex (DLPFC), attenuating knee osteoarthritis (KOA) pain [109]. Shenmen (HT7) acupuncture reduced cue-induced caudate nucleus activation, decreasing craving in alcohol use disorder [110].
7. Other Pathological Conditions: Acupuncture at BL13 (Danshu) and Zusanli (ST36) activated the anterior cingulate cortex, inferior temporal gyrus, and inferior parietal lobule, alleviating emotional distress and biliary colic in cholelithiasis patients [111, 112]. Zhongwan (CV12) stimulation decreased basolateral amygdala (BLA)-insula connectivity, improving symptom severity in functional dyspepsia (FD) [113]. Combinatorial Baihui (GV20) and Zusanli (ST36) treatment enhanced white matter integrity and cerebral blood flow in vascular dementia rat models [114].

**Table 3:** Dynamic neuromodulation of acupuncture on central brain regions and functional networks

Condition	Acupoints	Modulated brain regions/networks	Key effects	Ref
Hypertension	Taichong (LR3)	↑ Hypothalamus (ALFF/ReHo), ↑ brainstem-cerebellum connectivity	↑ autonomic regulation	[93]
	Taichong (LR3) + Yongquan (KI3)	↑ The prefrontal cortex (BA9,19,46), cingulate cortex (BA6,23,24,31), hypothalamic nuclei	↓ SBP/DBP	[94]
Migraine	Fengchi (GB20) + Hegu (LI4)	Modulated AMYG, INS, MCC; ↑ MTG/STG connectivity	↑ multisensory integration	[95]
	Verum acupuncture	↑ Right AMYG, left INS, vmPFC	↑ pain modulation network	[96]
	Taichong (LR3) + Hegu (LI4)	↑ Hypothalamic vlPAG-DLPFC coupling	Pain relief correlation	[97]
		Reversed dysfunction in AMYG, thalamus, ACC	↓ attack frequency	[98]
Insomnia	Baihui (GV20) + Shenmen (HT7)	↑ ACC-hippocampus-AMYG connectivity	↑ anxiety-related insomnia	[99]
	Shenmen (HT7) + Sanyinjiao (SP6) + Baihui (GV20)	↑ ALFF in cerebellar posterior lobe, brainstem, and frontoparietal regions	Modulated autonomic activity	[100]
	–	Normalized VMH-LOFC connectivity	↑sleep efficiency	[101]
Post-Stroke	Jin’s three-needle technique	↑ cerebellar posterior lobe, IPL, MTG	↑ motor control/sensory integration	[102]
	Hegu (LI4) + Zusanli (ST36)	Modulated SMN-DMN interaction	↑ limb functional recovery	[103]
	Neiguan (PC6) + Shuigou (DU26)	↑ Motor cortex-SMN connectivity	Neuroplastic reorganization	[104]

Cognitive Impairment	Baihui (GV20) + Shenmen (HT7) (electroacupuncture)	↑ Hippocampus-DMN connectivity	↓ diabetes-associated cognitive deficits	[105]
	Taichong (LR3) + Yongquan (KI1)	↑ Hippocampus-temporoparietal coupling	↓ AD symptoms	[106]
	Baihui (GV20) + Zusanli (ST36)	↑ Hippocampal volume, DMN synchronization	↓ subjective cognitive decline	[107]
Metabolic/Pain Disorders	Tianshu (ST25) + Zusanli (ST36)	Modulated hypothalamic NPY/AgRP & POMC/CART neurons	Appetite suppression	[108]
	Yanglingquan (GB34)	Rebalanced vlPAG-DLPFC connectivity	↓ knee osteoarthritis pain	[109]
	Shenmen (HT7)	↓ Caudate nucleus activation	↓ alcohol craving	[110]
Other Conditions	Feishu(BL13) + Zusanli (ST36)	↑ ACC, ITG, IPL	↓ emotional distress/biliary colic in cholelithiasis	[111, 112]
	Zhongwan (CV12)	↓ BLA-insula connectivity	↑ functional dyspepsia symptoms	[113]
	Baihui (GV20) + Zusanli (ST36) (rat model)	↑ White matter integrity, cerebral blood flow	Enhanced recovery in vascular dementia models	[114]

*ALFF* Amplitude of low-frequency fluctuation, *ReHo* Regional homogeneity, *SBP/DBP* Systolic/diastolic blood pressure, *AMYG* Amygdala, *INS* Insula, *MCC* Mid-cingulate cortex, *MTG/STG* Middle/superior temporal gyrus, *ACC* Anterior cingulate cortex, *LOFC* Lateral orbitofrontal cortex, *IPL* Inferior parietal lobule, *SMN/DMN* Sensorimotor/default mode network

The therapeutic effects of acupuncture are fundamentally mediated through dynamic neuromodulation of specific brain regions and functional networks. In hypertension and migraine pathologies, acupuncture elicits therapeutic responses by activating prefrontal cortical regions, cingulate areas, and hypothalamic nuclei to regulate autonomic balance and pain processing circuits. For insomnia and cognitive disorders, its efficacy primarily arises from enhanced functional integration within the default mode network (DMN), hippocampal formation, and limbic circuitry. Notably, acupuncture-mediated reorganization of the SMN proves critical for post-stroke functional recovery, while altered connectivity patterns in limbic structures (e.g., amygdala, insula) underlie its effectiveness in pain and emotion-related conditions. These findings collectively demonstrate acupuncture’s characteristic multi-target, multi-network synergistic mechanism, with scientific innovation rooted in bridging traditional acupoint theory with modern brain network paradigms—thereby establishing neuroimaging-based foundations for precision-targeted acupuncture interventions.

The therapeutic effects of acupuncture are fundamentally mediated through dynamic neuromodulation of specific brain regions and functional networks, building upon the peripheral neural signals initiated at acupoints and relayed through distinct pathways as detailed in Sects. “Multisystem interaction mechanisms underlying acupuncture activation” and “Activation of the peripheral nervous system (PNS): a necessary pathway for acupuncture efficacy”. In hypertension and migraine pathologies, acupuncture at acupoints like Taichong (LR3), Hegu (LI4), and Fengchi (GB20) elicits therapeutic responses by activating prefrontal cortical regions, cingulate areas, and hypothalamic nuclei to regulate autonomic balance and pain processing circuits. Crucially, acupuncture-mediated reorganization of the SMN, critical for functional recovery following peripheral activation at points like Neiguan (PC6) and Zusanli (ST36) (Sect. “Activation of spinal nerves” and “Activation of autonomic nerves”), proves essential for post-stroke rehabilitation. Similarly, altered connectivity in limbic structures (e.g., amygdala, insula) underpins acupuncture’s effectiveness in pain and emotion-related conditions, often following stimulation that engages spinal or autonomic pathways. Notably, for migraine relief, stimulation at Hegu (LI4) and Fengchi (GB20) engages vagal-mediated immune regulation (Sect. “Immune regulatory network”) or the trigeminal nerve pathway (Sect. “Activation of cranial nerves”), modulating central pain networks including the amygdala and insula. These findings collectively demonstrate acupuncture’s characteristic multi-target, multi-network synergistic mechanism across the “Skin-Brain Axis”, with scientific innovation rooted in bridging traditional acupoint theory with modern brain network paradigms—thereby establishing neuroimaging-based foundations for precision-targeted acupuncture interventions.

### The response of central brain regions as a necessary pathway for acupuncture efficacy

**Fig. 3:**
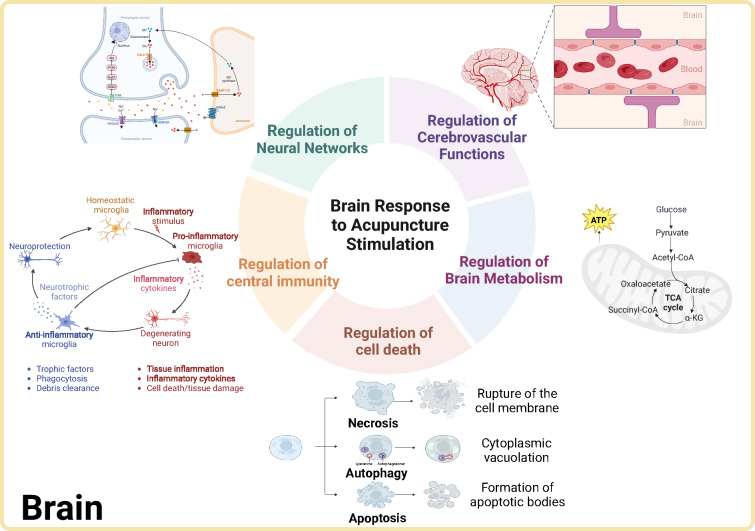
The diagram elucidates the central brain response to acupuncture stimulation. (Created with BioRender.com)

Ongoing advancements in contemporary research methodologies have elucidated intricate mechanisms underlying the brain’s response to acupuncture stimulation. This paper will expound upon five specific domains: the cerebral neural network, cerebrovascular function, brain metabolism, central immunity, and cell death, as detailed in Fig. 3.

1. Regulation of neural network: Acupuncture’s impact on these networks can be classified by its effects on neurons, synapses, and neural circuits. Neurons: EA at Zusanli (ST36) elevates levels of endogenous cannabinoids, such as 2-arachidonoylglycerol, and cannabinoid receptor 1, which reduces hyperactivation of pyramidal neurons and alleviates chronic pain [115]. Stimulation enhances GABAergic interneuron activity while inhibiting excitatory pyramidal neurons in the primary sensory cortex, thus mitigating neuropathic pain [116]. EA at Baihui (GV20) and Yintang (GV29) adjusts extracellular ATP levels in the prefrontal cortex, which improves depression-like behaviors in maternal separation models [117]. Moreover, stimulation at Zusanli (ST36) and Kunlun (BL60) activates dopaminergic projections from the ventral tegmental area to the nucleus accumbens, providing relief from both chronic pain and depressive-like symptoms in collagen-induced arthritis models [118]. EA at Shenting (GV24) enhances limbic-cortical functional connectivity, reducing amyloid-beta accumulation and improving memory in Alzheimer’s disease (AD) mouse models [119]. Synapses: EA at Baihui (GV20), Yintang (GV29), and Mingmen (GV4) activates the mTOR pathway in the prefrontal cortex, which suppresses autophagy, clears α-synuclein, and promotes synaptic protein expression, thereby alleviating depressive-like symptoms in Parkinson’s disease (PD) models [120]. Stimulation at Hegu (LI4), Baihui (GV20), and Yintang (GV29) enhances synaptic plasticity by modulating matrix metalloproteinases and inhibiting extracellular matrix deposition in the hippocampus, thus relieving lipopolysaccharide (LPS)-induced depression-like behaviors [121]. Acupuncture at Baihui (GV20), Hegu (LI4), Renzhong (GV26), and Zusanli (ST36) activates the BDNF/TrkB signaling pathway, facilitating recovery from synaptic damage in traumatic brain injury (TBI) models [122, 123]. Additionally, EA at Baihui (GV20) and Dazhui (GV14) activates miR-132, which improves hippocampal synaptic plasticity and mitigates cognitive deficits in sleep-deprived rats [124]. Neural circuit activation: EA at Neiguan (PC6) modulates cardiovascular autonomic functions through hypothalamic-brainstem pathways and interactions between the dorsal raphe nucleus and the ventral medullary choroid plexus, effectively treating atrial fibrillation [125]. Acupuncture at Shenmen (HT7) activates glutamatergic projections from the prefrontal cortex to the lateral habenula, suppressing cocaine-seeking behaviors [126]. Furthermore, EA at Shenmen (HT7) modulates the central amygdala and inhibits dopamine release in the nucleus accumbens, reducing methamphetamine-induced addictive behaviors in mice [127]. EA at Zhongwan (CV12) and Weishu (BL21) accelerates gastric emptying in mice with gastrointestinal dyskinesia, a process mediated by the suppression of GABA-ergic projections from the central amygdala to the dorsal vagal complex [128].
2. Regulation of cerebrovascular function: Cerebrovascular changes, including alterations in vascular permeability and angiogenesis, play a critical role in the development and progression of brain diseases. Increased vascular permeability can exacerbate inflammation and tissue damage, while angiogenesis facilitates tissue repair and regeneration [129, 130]. Vascular permeability: Acupuncture at Shuigou (GV26) and the bilateral Neiguan (PC6) acupoints have demonstrated the ability to rebalance autophagy and apoptosis in ischemic stroke models by modulating Beclin1/Bcl-2 complexes, reducing blood–brain barrier (BBB) permeability, and mitigating ischemic injury [131]. Acupuncture at Baihui (GV20) and Qubin (GB7) suppresses the rhoA/rho-associated coiled-coil containing kinase II (ROCK II)/myosin light chain 2 (MLC2) signaling pathway, thereby maintaining BBB integrity and facilitating functional recovery in experimental hemorrhagic stroke models [132]. Angiogenesis: EA at acupoints along the Yang meridian promotes angiogenesis in ischemic brain tissue of middle cerebral artery occlusion (MCAO) animal models through the upregulation of ephrin type-B receptor 4 (EphB4)/ephrin type-B receptor ligand 2(EphrinB2) and the activation of the sarcoma (Src) family kinase (Src) and phosphatidylinositol 3-kinase (PI3K) signaling pathway [133]. Additionally, EA at Shuigou (GV26) enhances the expression of the sonic hedgehog signaling pathway, fostering angiogenesis in damaged brain tissues in MCAO rat models [134, 135].
3. Regulation of brain metabolism: The brain, despite comprising only 2% of the body’s weight, utilizes about 20% of the body’s resting energy [136], making metabolic dysregulation a pivotal factor in the pathogenesis of brain disorders [137]. Mitochondrial energy metabolism: EA at Baihui (GV20), Yintang (GV29), and Zusanli (ST36) enhances mitochondrial respiratory enzyme activity, thereby increasing aerobic metabolism and attenuating brain injury in ischemia–reperfusion models [138]. Acupuncture at Baihui (GV20) and Shenting (GV24) effectively upregulates the expression of peroxisome proliferator-activated receptor gamma coactivator 1-alpha (PGC-1α), nuclear respiratory factor 1 (NRF-1), and mitochondrial transcription factor A (TFAM)-related mRNA and proteins, as well as boosts the activity of electron transport chain complexes I, IV, and V. This leads to elevated ATP levels, preservation of mitochondrial membrane potential, and mitigation of neuronal injury induced by acute cerebral ischemia [139]. Nutrient metabolism: Proteomics and metabolomics analyses indicate that acupuncture at Shenting (GV24), Wangu (BL10), and Fengchi (GB20) modulates key molecular pathways, particularly those involving arginine and energy metabolism, to alleviate migraine symptoms [140]. EA at Baihui (GV20) and Dazhui (GV14) enhances brain iron metabolism in a cerebral hemorrhage rat model, exerting neuroprotective effects by reducing iron-storage proteins and suppressing the expression of transferrin, transferrin receptors, and ferritin [141]. Similarly, acupuncture at Shuigou (GV26) during the acute phase of ischemic stroke helps maintain glutamate and GABA balance, contributing to cerebroprotection [142].
4. Regulation of central immunity: Brain inflammation is a critical factor in the progression of various neurological disorders, including immune-related conditions like multiple sclerosis, neurodegenerative diseases such as AD and PD, and brain injuries like stroke and TBI [143–145]. Abnormal glial cell polarization and excessive inflammatory cytokine production are common pathological features in encephalopathy [146]. Abnormal polarization of glial cells: Acupuncture at Shangxing (DU23) and Fengfu (GV16) exerts antidepressant effects by upregulating oxidative stress factors silent information regulator 2 (Sirtuin 1), nuclear factor erythroid 2-related factor 2(Nrf2), heme oxygenase-1(HO-1), and glutathione peroxidase 4(GPX4) in the hippocampal brain region of a rat depression model, while inhibiting the activation of microglia and astrocytes [147]. Transcutaneous electrical stimulation at Baihui (GV20) and Yintang (GV29) reduces ischemic stroke-induced brain damage by inhibiting inflammation, cell death, and microglial activation through suppression of the toll-like receptor 4 (TLR4)/myeloid differentiation primary response 88 (MyD88)/NF-κB signaling pathway [148]. Moreover, acupuncture inhibits the M1 phenotypic polarization of microglia and promotes M2 phenotypic polarization by targeting the TLR4/TRIF/MyD88 signaling pathway, thereby alleviating neurological dysfunction caused by TBI [149]. Massive enrichment of inflammatory cytokines: EA at Baihui (GV20) decreases the expression of pro-inflammatory cytokines (tumor necrosis factor-alpha (TNF-α), interleukin-1 beta(IL-1β), and IL-6) while increasing anti-inflammatory cytokines (interleukin-4 and interleukin-10) in the glial cells of an AD rat model [150]. Acupuncture at bilateral Zusanli (ST36) and Baihui (GV20) reduces inflammatory cells in the hippocampal brain region of rats with a chronic cerebral underperfusion model, improving cognitive dysfunction through activation of the α7 nicotinic acetylcholine receptor-mediated anti-inflammatory pathway [151]. EA at bilateral Fengchi (GB20) and Jiaji points reduce inflammatory cells, CD86 + /CD11b + immune cell infiltration in brain-injured tissues, and neurological impairment in rats with an acute cerebral hemorrhage model [152]. Furthermore, EA at Baihui (GV20) improves postoperative cognitive dysfunction in aged mice by increasing the expression of telomerase reverse transcriptase in the hippocampal brain region and enhancing its mitochondrial localization, thus reducing oxidative damage and neuroinflammation [153].
5. Regulation of cell death: Brain diseases are intrinsically linked to cell death, affecting both neurons and glial cells. The three primary mechanisms of cell death—apoptosis, autophagy, and ferroptosis—are crucial in the pathogenesis of various neurological conditions, such as AD and PD [154]. Cellular autophagy: Autophagy is essential for maintaining intracellular environmental stability [155]. Dysfunctional autophagy, critical for intracellular homeostasis, is a significant contributor to encephalopathy [156]. Research indicates that EA at Shuigou (GV26) inhibits excessive autophagy in ischemic brain tissues of MCAO model rats by activating the microRNA-34 (miR-34)/Wnt signaling pathway, thus providing cerebroprotection [157]. Following TBI, acupuncture accelerates the removal of damaged cellular structures on day 3 and suppresses excessive neuronal autophagy on days 7 and 14, thereby reducing neurological damage [158]. EA at Baihui (GV20) and Qubin (GB7) ameliorate neurological deficits in cerebral hemorrhage models by modulating the mTOR pathway and promoting neuronal autophagy [159]. Apoptosis: Apoptosis, a genetically programmed form of cell death characterized by cell shrinkage, chromatin condensation, and membrane integrity [160], is a pivotal factor in the pathogenesis of neurodegenerative diseases [161]. EA at Baihui (GV20) reduces cortical neuronal apoptosis in ischemic brain tissues of MCAO model rats by upregulating B-cell lymphoma 2 (Bcl-2) expression, decreasing caspase-3 levels, and enhancing histone acetylation, thereby reducing infarct volume [162]. Stimulation of Baihui (GV20) and Shenshu (BL23) inhibits hippocampal neuronal apoptosis and improves cognitive impairment in AD rat models [163] by downregulating apoptosis-related proteins (caspase-3 and caspase-9) and modulating circadian proteins clock and brain and muscle arnt-like protein 1 (Bmal1). EA at Baihui (GV20) and Shuigou (GV26) enhances recovery of learning and memory in MCAO model rats by increasing the uptake of nerve growth factor in the brain and reducing neuronal apoptosis in the hippocampal region [164]. Additionally, acupuncture at Shangxing (DU23) and Fengfu (GV16) exerts antidepressant effects by regulating the Nrf2/HO-1 signaling pathway, mitigating oxidative stress, and preventing neuronal apoptosis in a rat depression model [165]. Cellular iron death: Ferroptosis, an iron-dependent form of cell death induced by lipid peroxidation, is distinct from apoptosis and autophagy [154]. EA at Baihui (GV20) and Qubin (GB7) alleviates ferroptosis-induced lipid peroxidation injury following cerebral hemorrhage by downregulating miR-23a-3p and activating the sequestosome 1 (p62)/kelch-like ECH-associated protein 1 (Keap1)/Nrf2 antioxidant pathway, leading to the upregulation of ferritin heavy chain 1 and glutathione peroxidase 4 [166, 167]. EA at the Sishencong (EX-HN1) acupoint effectively suppresses iron-mediated ferroptosis in hippocampal neurons by elevating GPX4 and solute carrier family 7-member 11 (SLC7A11) expression, coupled with a decrease in 4-hydroxynonenal (4-HNE) levels, thereby ameliorating neuronal injury and enhancing spatial learning and memory capacities within the hippocampal regions of type 2 diabetic (T2DM) mice [168].

## Conclusion

In summary, the “Skin-Brain Axis” provides a robust neurobiological framework for understanding acupuncture. It delineates a multilevel process: initiated by stimulating cutaneous acupoints, propagated through precise peripheral neural pathways, and culminating in the targeted modulation of central neural networks. Mechanical stimulation of acupoints (e.g., Zusanli [ST36]) activates TRPV1/3/4 receptors and Aδ/C fibers, transmitting signals via DRG to key central nuclei (e.g., nucleus tractus solitarius, hypothalamus, amygdala). This integrated neural pathway dynamically modulates pain perception, autonomic balance (e.g., vagal-adrenal axis), and emotion-related networks (e.g., DMN-limbic interactions). Mechanical stimulation of Hegu (LI4) activates peripheral sensory pathways, transmitting signals via the vagus nerve to modulate macrophage α7nAChRs and suppress systemic pro-inflammatory cytokines (e.g., TNF-α). These signals further project centrally to dynamically reorganize functional connectivity between the SMN and DMN, enhancing post-stroke motor recovery. Mechanical stimulation of Neiguan (PC6) activates the median nerve, transmitting signals via C5-T1 DRG to spinal relay nuclei (e.g., lateral cervical, intermediolateral, and lateral spinal nuclei), which project to hypothalamic-brainstem pathways to modulate cardiovascular reflexes and suppress visceral sympathetic excitatory responses. This cross-scale “acupoint-peripheral-CNS” cascade explains both immediate effects (e.g., analgesia, blood pressure reduction) and sustained outcomes mediated by neuroplasticity and functional remodeling of brain networks.

Current studies have predominantly focused on isolated aspects of acupoint microenvironments, peripheral signaling, or central networks, with only a limited number of investigations holistically dissecting the cross-scale “acupoint-peripheral-CNS” signaling cascade. To address this gap, future studies must prioritize mechanistic clarity to transform acupuncture from empirical practice to precision neuromodulation. Critical steps including developing real-time fMRI or wearable biosensing technologies to establish “acupuncture stimulus parameter-effect gradient” models using multimodal neuroimaging and transcriptomics to systematically compare stimulation modalities (e.g., electroacupuncture, manual needling, laser acupuncture, transcutaneous electrical stimulation). Meanwhile, artificial intelligence-powered spatiotemporal maps of acupoint-CNS interactions will guide clinical acupoint selection and optimize stimulation parameters based on real-time physiological feedback (e.g., pain thresholds, and autonomic tone). Furthermore, applying single-cell sequencing, neural circuit mapping, and multimodal imaging to delineate acupoint-specific effects on brain networks and molecular pathways. Moreover, constructing spatiotemporal signaling maps to identify critical therapeutic nodes in the acupoint-CNS axis. Together, the “Skin-Brain Axis” framework bridges traditional acupuncture theory and modern neurobiology, offering a paradigm for maximizing therapeutic efficacy and catalyze the convergence of traditional medicine with contemporary neuroscience.

## Data Availability

No data was used for the research described in the article.

## Abbreviations

ACC: Anterior cingulate cortex
AD: Alzheimer’s disease
Aδ: Aδ fibers
Aβ: Aβ fibers
ALFF: Amplitude of low-frequency fluctuation
AMYG: Amygdala
ATP: Adenosine triphosphate
Bcl-2: B-cell lymphoma 2
BDNF: Brain-derived neurotrophic factor
BNST: Bed nucleus of the stria terminalis
CB2R: Cannabinoid type 2 receptor
C-fibers: C fibers
CGRP: Calcitonin gene-related peptide
CNS: Central nervous system
CTB: Cholera toxin subunit B
CXCL12: Chemokine (C-X-C motif) ligand 12
DBP: Diastolic blood pressure
DMN: Default mode network
DRG: Dorsal root ganglion
fMRI: Functional magnetic resonance imaging
GABA: Gamma-aminobutyric acid
HPA axis: Hypothalamic–pituitary–adrenal axis
HRP: Horseradish peroxidase
IL-4/IL-10: Interleukin-4/interleukin-10
INS: Insula
IPL: Inferior parietal lobule
MAPK: Mitogen-activated protein kinase
MCC: Mid-cingulate cortex
MTG/STG: Middle temporal gyrus/superior temporal gyrus
NAU: Neural acupuncture unit
NF-κB: Nuclear factor kappa-light-chain-enhancer of activated B cells
NO: Nitric oxide
NPY: Neuropeptide Y
PGE2: Prostaglandin E2
PNS: Peripheral nervous system
PRV: Pseudorabies virus
ReHo: Regional homogeneity
ROCK: Rho-associated protein kinase
SBP: Systolic blood pressure
SMN: Sensorimotor network
T2DM: Type 2 diabetes mellitus
TG: Trigeminal ganglion
TNF-α: Tumor necrosis factor-alpha
TRPV1/3/4: Transient receptor potential vanilloid 1/3/4
vlPAG: Ventrolateral periaqueductal gray
VMH: Ventromedial hypothalamic nucleus
IML: Intermediolateral nucleus
LCN: Lateral cervical nucleus
LSN: Lateral spinal nucleus
